# Molecular Basis for Antigenic Diversity of Genus *Betanodavirus*

**DOI:** 10.1371/journal.pone.0158814

**Published:** 2016-07-20

**Authors:** Valentina Panzarin, Anna Toffan, Miriam Abbadi, Alessandra Buratin, Marzia Mancin, Stine Braaen, Christel Moræus Olsen, Luca Bargelloni, Espen Rimstad, Giovanni Cattoli

**Affiliations:** 1 OIE Reference Laboratory for Viral Encephalopathy and Retinopathy, Department of Comparative Biomedical Sciences, Istituto Zooprofilattico Sperimentale delle Venezie, Legnaro (PD), Italy; 2 Department of Comparative Biomedicine and Food Science, University of Padua, Legnaro (PD), Italy; 3 Department of Food Safety and Infection Biology, Norwegian University of Life Sciences, Oslo, Norway; National Cheng Kung University, TAIWAN

## Abstract

Betanodaviruses are the causative agents of viral nervous necrosis (VNN), a devastating disease for the Mediterranean mariculture. Four different betanodavirus species are recognized, Striped jack-, Redspotted grouper-, Tiger puffer-, and Barfin flounder nervous necrosis virus (SJNNV, RGNNV, TPNNV and BFNNV), but there is little knowledge on their antigenic properties. In order to describe the serological relationships among different betanodavirus genotypes, serum neutralization assays were performed using rabbit polyclonal antisera against eight fish nodaviruses that cover a wide species-, temporal-, spatial- and genetic range. The results indicate that the SJNNV and RGNNV are antigenically distinct, constituting serotypes A and C, respectively. The TPNNV and BFNNV, the latter representing cold-water betanodaviruses, are antigenically related and cluster within serotype B. The reassortant viruses RGNNV/SJNNV and SJNNV/RGNNV group within serotypes A and C, respectively, indicating that the coat protein encoded by RNA2 acts as major immunoreactivity determinant. Immunostaining of *in vitro* expressed wild type and chimeric capsid proteins between the RGNNV and the SJNNV species indicated that the C-terminal part of the capsid protein retains the immunoreactive portion. The amino acid (aa) residues determining RGNNV and SJNNV antigenic diversity were mapped to aa residues 217–256 and aa 257–341, respectively. Neutralization of reverse genetics derived chimeric viruses indicated that these areas determine the neutralizing epitopes. The data obtained are crucial for the development of targeted serological tests for the diagnosis of VNN, and informative for development of cross-protective vaccines against various betanodavirus genotypes.

## Introduction

Decreasing wild stocks combined with increasing consumer demand for fish have contributed to the rapid expansion of the aquaculture production in the last decades, with a global yield of approximately 47 million tons in 2013 [1]. However, in industrialized farming the fish are kept at high densities and thus vulnerable to various infectious diseases, which represent a major threat for the sustainability of aquaculture production [2]. Viral nervous necrosis (VNN) (synonymous viral encephalopathy and retinopathy—VER) is an important infectious disease of farmed fish, and is caused by betanodaviruses. VNN represents a main bottleneck for the farming of marine finfish, and causes disease outbreaks in species such as European sea bass (*Dicentrarchus labrax*), gilthead sea bream (*Sparus aurata*), Senegalensis sole (*Solea senegalensis*), barramundi (*Lates calcarifer*) and groupers (*Epinephelus spp*.) [3–14]. Furthermore, VNN also impedes the introduction of new marine fish species as alternatives for farming [15]. Fish nodaviruses have global distribution and can infect a large number of different fish species [16]. They have a bi-segmented, single-stranded, positive sense RNA genome. The genomic segment RNA1 of approximately 3.1 Kb encodes the viral polymerase, otherwise known as protein A. The RNA1 molecule gives rise also to the subgenomic transcript RNA3 (0.4 Kb) which is translated in a +1 reading frame relative to the viral polymerase and encodes protein B2, a suppressor of cellular RNA interference (RNAi). The RNA2 segment of 1.4 Kb encodes the coat protein (CP) [17–20]. The structure of recombinant virus-like particles (VLPs) of a nodavirus from *Epinephelus malabaricus*, as observed by electron cryomicroscopy, suggests that the portion between amino acids (aa) 217 and 308 of the coat protein putatively forms a protrusion in the outer part of the capsid [21]. In a more recent study, the crystal structure of a grouper VLP was resolved, and three major domains were identified: the N-terminal arm (aa 34–51) and the shell domain (S-domain) (52–213) connected through the linker region (aa 214–220) to the protrusion domain (P-domain) (221–338) [22]. The P-domain harbors the putative host-specificity determinants of betanodaviruses [23] and the encoding nucleotide sequence includes the T4 variable region that can be utilized to classify fish nodaviruses into four distinct genotypes, namely RGNNV, SJNNV, TPNNV and BFNNV [24]. The BFNNV group also contains the psychrophilic betanodavirus strains of Atlantic cod (*Gadus morhua*) and Atlantic halibut (*Hippoglossus hippoglossus*) [25,26]. Interestingly, the genetic diversity among fish nodaviruses is further increased by reassortment of genomic segments, as observed between the RGNNV and the SJNNV genotypes, resulting in the reassortant strains RGNNV/SJNNV and SJNNV/RGNNV [11,27,28]. Although diverse fish nodavirus genotypes possess a number of common antigenic determinants [29,30], the genetic variety among the four betanodavirus genogroups reflects the differences in their immunoreactivity. By means of indirect fluorescent antibody tests (IFAT) and seroneutralization assays (SN), Mori and colleagues classified fish nodaviruses into three different serogroups. Serotype A consists of SJNNV strains, serotype B correspond to the TPNNV genotype, and serotype C comprises the RGNNV and the BFNNV species [29]. However, this classification is mainly based on viral isolates from Asia and neglects cold-water strains as well as reassortants, which were described only after the classification was made.

More information on the serological characteristics of these viruses are needed to develop adequate control strategies against VNN. In order to provide a comprehensive and up to date overview of the immunological properties of betanodaviruses, the present study aimed to cover the knowledge gap of the serological reactivity of betanodaviruses from the Mediterranean, cold-water strains and reassortant viruses. Furthermore, to better understand the molecular traits responsible for betanodavirus immunoreactivity, the immunostaining of wild-type and chimeric coat proteins harboring amino acid residues of the RGNNV and the SJNNV viruses was performed, and wild-type and chimeric betanodaviruses synthetized through the reverse genetics (RG) were serologically characterized. Data obtained are relevant for the implementation of targeted serological diagnosis of betanodaviruses and for providing useful information for the future development of vaccines protective against different betanodavirus genotypes.

## Results

### Serological classification of fish nodaviruses

Serum neutralization titres obtained by testing the rabbit antisera produced against each betanodavirus antigen are given in supplementary material (S1 Table). High neutralizing titres, ranging from 1:1280 to 1:40960, were obtained when testing rabbit antisera against the homologous antigens. Sera specificity was tested also against other fish viruses such as viral haemorrhagic septicaemia virus (VHSV) and infectious haematopoietic necrosis virus (IHNV). No cross-reactions were observed (S1 Table).

The serological relationships among betanodaviruses with different genomes are described by the 1/r value, defined as the reciprocal of the geometric mean titre (GMT) of the two ratios obtained with the heterologous viruses and the homologous sera [31]. Larger 1/r values indicate greater serological differences between strains (Table 1). By the use of a cut-off value of 10 [32], viruses analysed in the present study can be divided into three different serological groups called A, B and C, in compliance with the nomenclature previously established by Mori et al. [29]. In detail, strains 484.2.2009 (SJNNV) and 367.2.2005 (RGNNV/SJNNV) form serogroup A (1/r = 2.07), while viruses 283.2009 (RGNNV) and 389/I96 (SJNNV/RGNNV) cluster within serogroup C (1/r = 2.54). The serotyping of the reassortant strains RGNNV/SJNNV and SJNNV/RGNNV has led to the observation that the genetic type of the coat protein gene actually drives the immunoreactivity of these viruses. Differently from the serological categorization proposed by Mori and collaborators [29], strain JFIwa98 (BFNNV) does not belong to serogroup C, but can be classified as part of serogroup B together with strains TPKag93 (TPNNV), SK-07 1324 (BFNNV) and Ah95NorA (BFNNV) (1/r values ranging between 0.65 and 3.95). However, it must be mentioned that most of the 1/r values estimated among members of serogroups B and C are slightly above the threshold (i.e. 10.56–19.70), thus suggesting a certain level of cross-reactivity among genotypes RGNNV, SJNNV/RGNNV, TPNNV and BFNNV.

**Table 1:** 1/r values and Spearman coefficients among different betanodavirus isolates.

		1/r*							
		283.2009	389/I96	484.2.2009	367.2.2005	JFIwa98	TPKag93	SK-07 1324	Ah95NorA
Spearman correlation coefficient**	283.2009		**2.54**	53.82	59.71	19.70	17.75	10.56	16.95
	389/I96	**0.87**		173.99	99.93	47.43	17.97	23.72	17.16
		**<0.0001**							
		**(32)**							
	484.2.2009	**0.50**	0.33		**2.07**	84.45	34.90	51.98	33.90
		**0.0038**	0.0688						
		**(31)**	(31)						
	367.2.2005	**0.50**	0.33	**0.98**		119.43	49.35	48.50	59.03
		**0.0053**	0.0728	**<0.0001**					
		**(29)**	(29)	**(29)**					
	JFIwa98	0.25	0.25	-0.20	-0.21		**0.65**	**2.14**	**3.89**
		0.1597	0.1592	0.2609	0.2580				
		(32)	(32)	(31)	(29)				
	TPKag93	0.19	-0.14	**-0.40**	**-0.40**	**0.35**		**0.75**	**1.26**
		0.2728	0.4353	**0.0237**	**0.0314**	**0.0492**			
		(32)	(32)	**(31)**	**(29)**	**(32)**			
	SK-07 1324	0.34	**0.40**	-0.02	-0.02	**0.55**	0.24		**3.95**
		0.0517	**0.0203**	0.8892	0.9161	**0.0010**	0.1772		
		(32)	**(32)**	(31)	(29)	**(32)**	(32)		
	Ah95NorA	**0.46**	0.25	**0.49**	**0.56**	0.31	-0.20	-0.01	
		**0.0073**	0.1577	**0.0043**	**0.0015**	0.0751	0.2616	0.9185	
		**(32)**	(32)	**(31)**	**(29)**	(32)	(32)	(32)	

*Serological relationships of genetically different betanodaviruses expressed as 1/r values. Values < 10 (in bold) indicate that two viruses belong to the same serogroup.

**Spearman correlation coefficients between viruses and *p*-value. The number of observations is reported between brackets. Statistically significant correlations (*p*-value < 0.05) are highlighted in bold.

### Statistical analyses

Spearman coefficients are reported in Table 1. A very strong correlation is observed between viruses of serotype A (484.2.2009 and 367.2.2005) and between viruses of serotype C (283.2009 and 389/I96). Strain 283.2009 is moderately correlated also with viruses 484.2.2009 and 367.2.2005. Viruses belonging to serotype B correlate with each other, and correlate also with strains of serotypes A and C. In detail, virus JFIwa98 shows a weak correlation with strain TPKag93 and a moderate correlation with virus SK-07 1324. This latter is also moderately correlated with virus 389/I96. Strain Ah95NorA shows a moderate correlation with viruses belonging to serotypes A and C (484.2.2009, 367.2.2005 and 283.2009). Finally, strain TPKag93 is significantly uncorrelated with viruses of serotype A (484.2.2009 and 367.2.2005).

**Fig 1:**
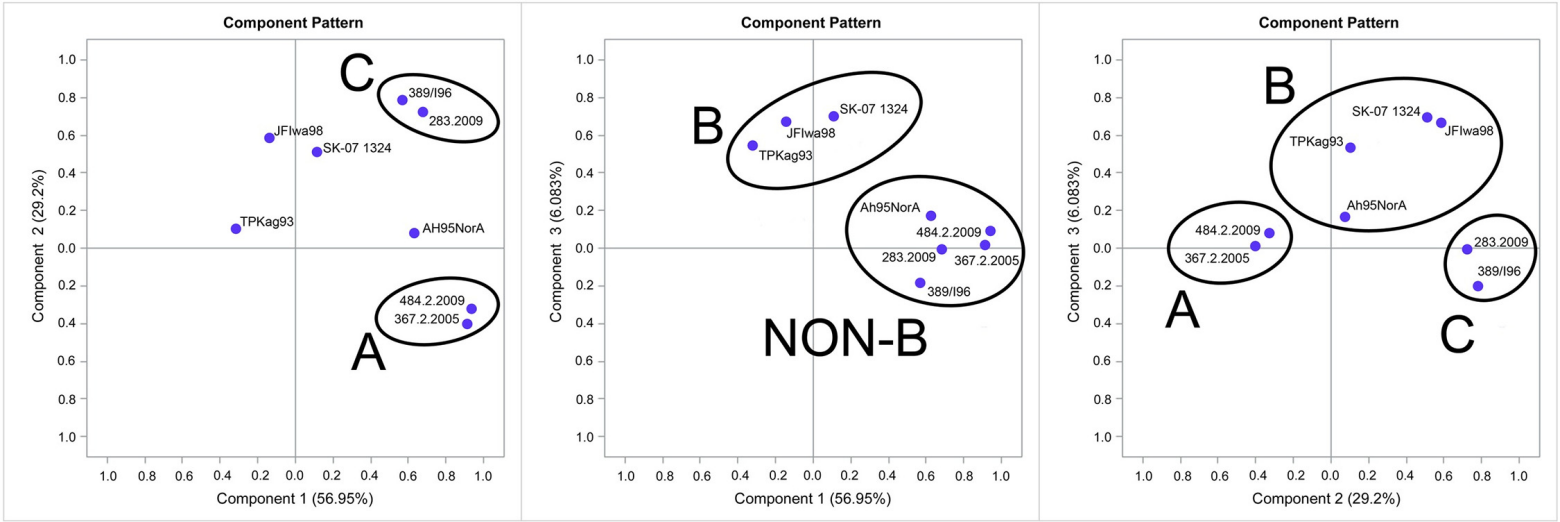
Clustering of viruses by pattern plots of the principal component (PC). The principal components analysis is based on the serum neutralization titres expressed as GMT of the isolates tested. In each graphic, the three PCs are plotted one against the other. Viruses are grouped into three different clusters named A, B and C.

The scree plot and the eigenvalue obtained by the PCA analysis indicate that the first three principal components account for a meaningful amount of variance. The total explained variability by the first 3 PCs is 92% (56.95% for PC1; 29.2% for PC2; 6.08% for PC3) (S1 Fig). Viruses RGNNV, SJNNV, RGNNV/SJNNV and SJNNV/RGNNV identify the first component, while the second component arranges viruses into two different groups named A (SJNNV, RGNNV/SJNNV) and C (RGNNV and SJNNV/RGNNV). Viruses JFIwa98, TPKag93, SK-07 1324 and Ah95NorA identify the third principal component and cluster together within group B (Fig 1).

### Serological typing of field isolates

To further evaluate the capability of the SN assay to recognize the serotype of unidentified samples, 11 field isolates were used as antigens and tested with the raised antisera. The neutralization titres obtained are shown in Table 2. The field strains clustered in the expected serotypes in accordance to their genotype. In detail, strains 132.2005, 82/I07 and 250.1.2009 (reassortants RGNNV/SJNNV) were classified as serotype A, while isolates 512.2000, 390.3.2003, 80.1.5.2005, 498.2.2005, 550.2.2005, 320.1.2009, 396.3.2011 and E.marginatus/I/35-1/Dec13 (RGNNV) fell within serotype C.

**Table 2:** Serological typing of field isolates through serum neutralization tests.

	**Rabbit antisera**							
**Field isolates (genotype)**	Serum anti-283.2009	Serum anti anti-389/I96	Serum anti-484.2.2009	Serum anti-367.2.2005	Serum anti-JFIwa98	Serum anti-SK-07 1324	Serum anti-TPKag93	Serum anti-Ah95NorA
512.2000 (RGNNV)	1:10240	1:640	1:10	1:320	1:10	1:40	1:160	1:10
390.3.2003 (RGNNV)	1:20480	1:1280	1:10	1:640	1:160	1:160	1:160	1:10
80.1.5.2005 (RGNNV)	1:20480	1:1280	1:40	1:640	1:160	1:160	1:160	1:20
498.2.2005 (RGNNV)	1:10240	1:640	1:10	1:320	1:80	1:80	1:40	1:80
550.2.2005 (RGNNV)	1:10240	1:640	1:40	1:640	1:40	1:160	1:80	1:80
320.1.2009 (RGNNV)	1:10240	1:2560	1:80	1:640	1:80	1:160	1:40	1:10
396.3.2011 (RGNNV)	1:10240	1:2560	1:160	1:1280	1:80	1:80	1:2560	1:20
E.marginatus/I/35-1/Dec13 (RGNNV)	1:5120	1:1280	1:160	1:640	1:40	1:40	1:640	1:10
132.2005 (RGNNV/SJNNV)	1:160	1:80	1:10240	1:20480	1:10	1:10	1:20	1:80
82/I07 (RGNNV/SJNNV)	1:640	1:160	1:2560	1:5120	1:10	1:10	1:10	1:320
250.1.2009 (RGNNV/SJNNV)	1:160	1:80	1:10240	1:20480	1:10	1:10	1:20	1:640

### Recombinant capsid proteins expression and immunostaining

**Fig 2:**
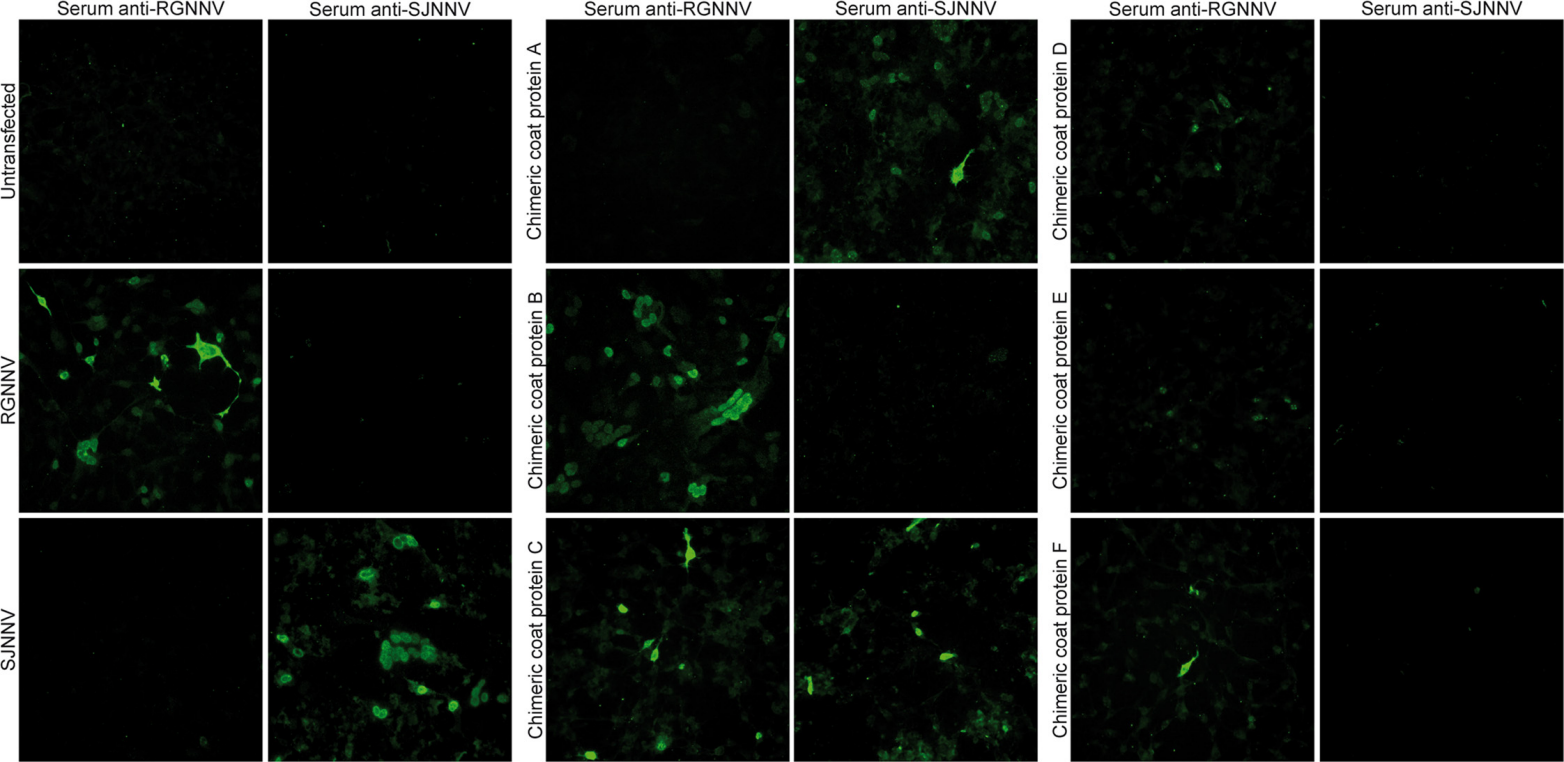
**EPC cells expressing RGNNV, SJNNV and chimeric capsid proteins A, B, C, D, E and F.** Cells were immunostained with serum anti-283.2009 (RGNNV) and anti-484.2.2009 (SJNNV). Images were taken at 20x magnification.

RGNNV, SJNNV and chimeric capsid proteins A, B, C, D, E and F were successfully expressed in EPC cells, and antigenically characterized with anti-RGNNV and anti-SJNNV (Fig 2). A moderate level of cytotoxicity was observed in EPC cells overexpressing recombinant capsid proteins, while non-transfected cells appeared unaltered (data not shown). Staining using the anti-RGNNV serum was observed for cells transfected with RGNNV CP, and chimeric capsid proteins B, C and F. RGNNV and chimeric capsid protein C and F had both nuclear and cytosolic staining, while chimeric protein B showed only nuclear staining. A significantly lower transfection efficiency was observed for chimeric protein F. The RNA2 sequences encoding for capsid proteins RGNNV, B, C and F share the RGNNV nucleotide sequence 676–795 (aa 217–256). In EPC cells expressing chimeric capsid proteins D and E, anti-RGNNV stained weakly a few nuclei with a granular pattern. Serum anti-SJNNV recognized SJNNV CP and chimeric capsid protein A and C, a granular pattern in some nuclei could be seen in A. The RNA2 sequences encoding for these proteins contain the SJNNV nucleotide sequence 796–1023 (aa 257–341). No fluorescent signal was observed in EPC cells stained with serum anti-SJNNV expressing capsid proteins RGNNV, B, D, E and F. Notably, chimeric capsid protein C was recognized by both sera anti-RGNNV and anti-SJNNV.

### Recovery and characterization of reverse genetics viruses

**Fig 3:**
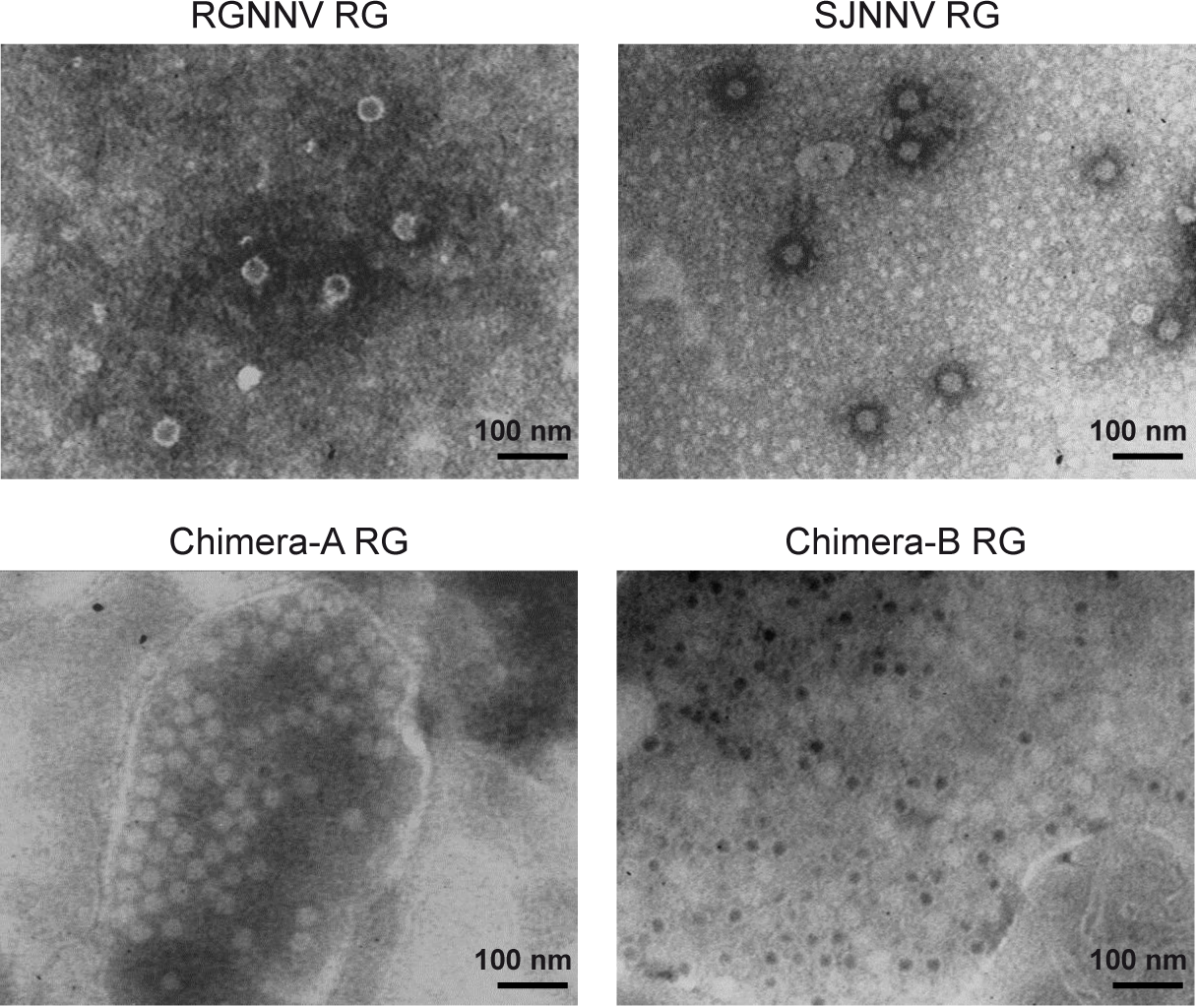
Negative staining TEM of recombinant viruses obtained through the reverse genetics technology. Pictures were taken at 28x magnification.

The generation of progeny viruses from infectious transcripts was determined by CPE formation and TEM observation. At 24 hours post transfection (h.p.t.), typical foci of rounded, granular and vacuolated cells were observed in all samples. At 48 h.p.t., cell monolayers were completely disrupted. E-11 cells inoculated with Lipofectamine® MessengerMAX™ Transfection Reagent only, showed no cytotoxicity. Only the RGNNV RG, the SJNNV RG, chimera-A RG and chimera-B RG replicated and were able to produce typical CPE after passage to new E-11 monolayers. When subjected to TEM, virions compatible in both size and shape with betanodavirus particles were observed for these samples only (Fig 3).

The recovered RG viruses and the wild-type RGNNV and SJNNV were subjected to SN with anti-RGNNV and anti-SJNNV (Table 3). Viruses RGNNV wild-type (283.2009) and RGNNV RG were specifically neutralized by anti-RGNNV (1:10240 and 1:1280, respectively), while their SN with anti-SJNNV was negligible (1:10). On the other hand, anti-SJNNV strongly neutralized SJNNV wild-type (484.2.2009) (1:20480) and SJNNV RG (1:5120/1:10240), however, these were also slightly neutralized by anti-RGNNV (1:320/1:640 and 1:640, respectively). Remarkably, data obtained from the characterization of chimera-A RG and chimera-B RG indicate that their serological profiles are primarily determined by the C-terminus of their coat proteins. Indeed, chimera-A RG was strongly neutralized by anti-SJNNV, with neutralizing titre similar to that obtained for the SJNNV wild type (1:20480). Chimera-A RG was also neutralized by anti-RGNNV, but at significantly lower extent (1:2560/1:5120). Conversely, chimera-B RG was strongly neutralized by anti-RGNNV, with neutralizing titre identical to that of the RGNNV wild type (1:10240), while it was barely neutralized by anti-SJNNV (1:160/1:320).

**Table 3:** Serum neutralization tests of wild type and recombinant viruses.

	**Serum anti-283.2009**	**Serum anti-484.2.2009**
**283.2009 wild-type**	1:10240	1:10
**484.2.2009 wild-type**	1:320/1:640	1:20480
**RGNNV RG**	1:1280	1:10
**SJNNV RG**	1:640	1:5120/1:10240
**Chimera-A RG**	1:2560/1:5120	1:20480
**Chimera-B RG**	1:10240	1:160/1:320

Wild type strains 283.2009 (RGNNV) and 484.2.2009 (SJNNV), and recombinant viruses RGNNV RG, SJNNV RG, chimera-A RG and chimera-B RG were subjected to serum neutralization using rabbit hyperimmune sera anti-283.2009 and anti-484.2.2009. SNs were carried out in four replicates. Results are expressed as neutralizing titres.

## Discussion

The study of the immunoreactivity and the immunogenicity of fish pathogens is required for developing serological diagnostic tests and for designing vaccines. Information on the antigenic properties is particularly scarce for the fish nodaviruses, and currently there is only one paper providing serological characterization of different betanodavirus genotypes, that originated mostly from Asia [29]. The serological classification proposed was used in our study but cold-water strains, the newly emerged reassortants and viruses from the Mediterranean basin were implemented. Our results showed that betanodaviruses can be classified into 3 different serogroups, namely A, B and C, in conformity with the nomenclature previously established. Serotype A comprises SJNNV and RGNNV/SJNNV viruses, while serotype C consists of RGNNV and SJNNV/RGNNV strains. Differently from what previously reported, our data show that the BFNNV genotype clusters together with the TPNNV virus and the cold water isolates within serotype B. In their work, Mori and colleagues had used a threshold of 20 for the 1/r index to establish whether two different viruses belonged to the same serogroup. According to this criterion, they categorized the BFNNV genotype within serotype C, although they had also observed an exceptional cross-reactivity with the TPNNV genotype (1/r = 16) [29]. In the present study, we have set up a lower threshold for the 1/r index (i.e. 10), as adopted by Hill and Way [32] for the serological categorization of aquatic *Birnaviruses*. This choice was further supported by the PCA that clearly identified 3 clusters of viruses: A (SJNNV, RGNNV/SJNNV), B (BFNNV, TPNNV) and C (RGNNV, SJNNV/RGNNV) (Fig 1). However, it must be mentioned that some of the 1/r values calculated among isolates of serogroup B are slightly above the threshold (10 < 1/r < 20), and the Spearman coefficients calculated for these viruses highlight for some of them a weak/moderate correlation with strains belonging to serotypes A and C. Taken together, these data suggests that viruses belonging to serotype B are partially cross-reactive with viruses belonging the other serotypes.

The antigenic differences between the RGNNV and the SJNNV genotypes have been assessed by several authors [29,30,33–35]. Consistent with their assumptions, we found that there is little or no cross-reactivity between betanodaviruses of serogroups A and C, as confirmed by the high 1/r values and statistical analyses. Furthermore, our data indicate that the serotype of the RGNNV/SJNNV and SJNNV/RGNNV reassortants is determined by the genotype of the donor for the RNA2 segment, i.e. the coat protein gene is the major genetic determinant of betanodavirus immunoreactivity. Tang et al. [21] reconstructed the 3D structure of the malabaricus grouper nervous necrosis virus VLPs, suggesting that residues 217–388 form a protruding domain located at the outer surface of the capsid. Similarly, Chen et al. [22] described the crystal structure of GNNV-LP and identified a protrusion domain (aa 214–388) putatively involved in the calcium-mediated trimerization of the capsid proteins during the inner phases of betanodavirus capsid assembly. Besides, the authors observed that a number of hypervariable regions of the P-domain overlap with functional regions implicated in cell receptor binding and species tropism [23]. Accordingly, we hypothesized that the protrusion domain might also be involved in other host-pathogen interaction processes, such as antibody recognition. Interestingly, data obtained from the immunostaining of chimeric coat proteins A and B demonstrate that residues from aa 217 to the C-terminus retain the immunoreactive portion of the RGNNV and the SJNNV capsid proteins, in concordance with the 3D structural models proposed by Tang et al. and Chen et al. [21,22]. It is also noteworthy that SN tests carried out for chimera-A RG and chimera-B RG indicate that the epitopes comprised between aa 217 and the C-terminus of their coat proteins contain the neutralizing epitopes. Remarkably, the immunostaining patterns of EPC cells expressing the various chimeric capsid proteins indicate that the immunoreactive residues of the RGNNV and the SJNNV genotypes are located at different positions of the coat protein sequence. Proteins retaining the RGNNV aa residues 217–256 were recognized by anti-RGNNV, while proteins containing the SJNNV aa sequence 257–341 reacted with anti-SJNNV (Fig 2). Immunoblotting partial coat proteins of the four known species with SJNNV specific MAbs, Nishizawa et al. [30] pinpointed the PAN_254-256_ motif of the SJNNV capsid protein as a candidate neutralizing epitope for this genotype. In the present study, the absence of staining with serum anti-SJNNV of chimeric proteins D and E, which contain the PAN_254-256_ motif, contradicts this finding. Such a result might be due to the fact that the PAN_254-256_ motif is a linear epitope, and therefore it might not be accessible to antibody recognition using serum anti-484.2.2009. On the other hand, Iwamoto and colleagues [36] reported that putative bulging positions in the 236–338 region of the SJNNV and the RGNNV CPs show noticeable differences in their surface probability scores, suggesting the presence of structural variations between the capsid proteins of these two genotypes. This observation might corroborate the hypothesis that the immunoreactive epitopes of the RGNNV and the SJNNV genotypes are situated at different positions in the coat protein sequence. Interestingly, Ito and colleagues [23] identified the nucleotide regions 694–758 for the RGNNV and 695–765 for the SJNNV as the molecular determinants controlling species tropism. Notably, the RNA2 sequence regulating the RGNNV host specificity is comprised within the nucleotide region 675–795 determining also the immunoreactivity for this genotype. On the contrary, the capsid protein portions involved in host tropism and antibody recognition of the SJNNV are encoded by different nucleotide regions (nt 695–765 and nt 796–1023, respectively). It cannot be ruled out that the structural and functional differences observed between the RGNNV and the SJNNV might be responsible for the diverse phenotypes in terms of species specificity and pathogenicity observed for these genotypes.

Data obtained from the immunostaining of chimeric coat proteins were supported by the results of the serological neutralization test of the reverse genetics derived viruses, at least for the RG strains tested in this study. We were unable to obtain chimera-C RG, chimera-D RG, chimera-E RG and chimera-F RG, despite we observed complete CPE at 48 hpt for these viruses. It is noteworthy that the chimeric constructs C, D, E and F present a junction between the RGNNV and the SJNNV sequences at the nucleotide position 795. We hypothesize that such a genetic manipulation might have generated defective particles unable to replicate due to the interruption of genetic signatures that are critical for viral viability, for instance recruiting signal for the formation of the replication complex, packaging signal, capsid assembly signal, protein localization signal, etc. Further investigations are required to explore the role of this for betanodavirus biology and fitness.

In summary, in the present study we have characterised some determinants responsible for betanodavirus immunoreactivity including an updated overview of the serological relationships among different genotypes. The results show that serological characterization is a good alternative to genetic typing of betanodaviruses. Importantly, field isolates were grouped consistently by SN test and genotyping, thus validating the SN test as a specific method for routine diagnosis and typing. Indeed, the low cross-reactions among different betanodavirus serotypes indicate that the SN test could be useful for virus characterization in areas where more than one betanodavirus genotype is circulating, such as the Mediterranean basin [27]. Furthermore, the serum neutralization test described in the present study has a potential for being applied as a screening method to test newly introduced broodstock into hatcheries, thus avoiding the suppression of animals of high commercial value, or to assess the protection elicited by immunization procedures. Indeed, although early diagnosis and biosafety measures play a primary role in VNN control, vaccination remains the elective prophylactic method for a sound long-term sustainable economic, environmental and ethical basis. Our data indicate that monovalent vaccines will not be sufficient to obtain protection, which may be important information for future development of vaccines against VNN caused by any betanodavirus genotype. In this view, the cross-reaction of chimeric protein C with serum anti-RGNNV and anti-SJNNV observed in the present study is of particular interest for the development of a candidate wide-spectrum vaccine intended for administration in adult fish as well as in juveniles before they are moved from the hatchery to sea cages.

## Materials and Methods

### Cell lines

E-11 fibroblasts (directly supplied by the European Collection of Authenticated Cell Cultures, ECACC), cloned from the SSN-1 cell line (striped snakehead, *Ophicephalus striatus*) [37] were grown at 25°C in L-15 medium (Leibovitz) (Sigma-Aldrich) containing 10% FCS, L-Glutamine (2mM) and antibiotics (100 IU/mL penicillin, 100 μg/mL streptomycin and 0.25 μg/mL amphotericin B) [38].

The epithelioid cell line (EPC) (Sigma-Aldrich, catalogue no. 93120820) from epithelioma of Fathead minnow (*Pimephales promelas*) [39] was maintained at 20°C in L-15 medium (Leibovitz) (Life Technologies) supplemented with 10% FCS and gentamycin-sulfate (50 μg/mL).

### Betanodaviral strains

**Fig 4:**
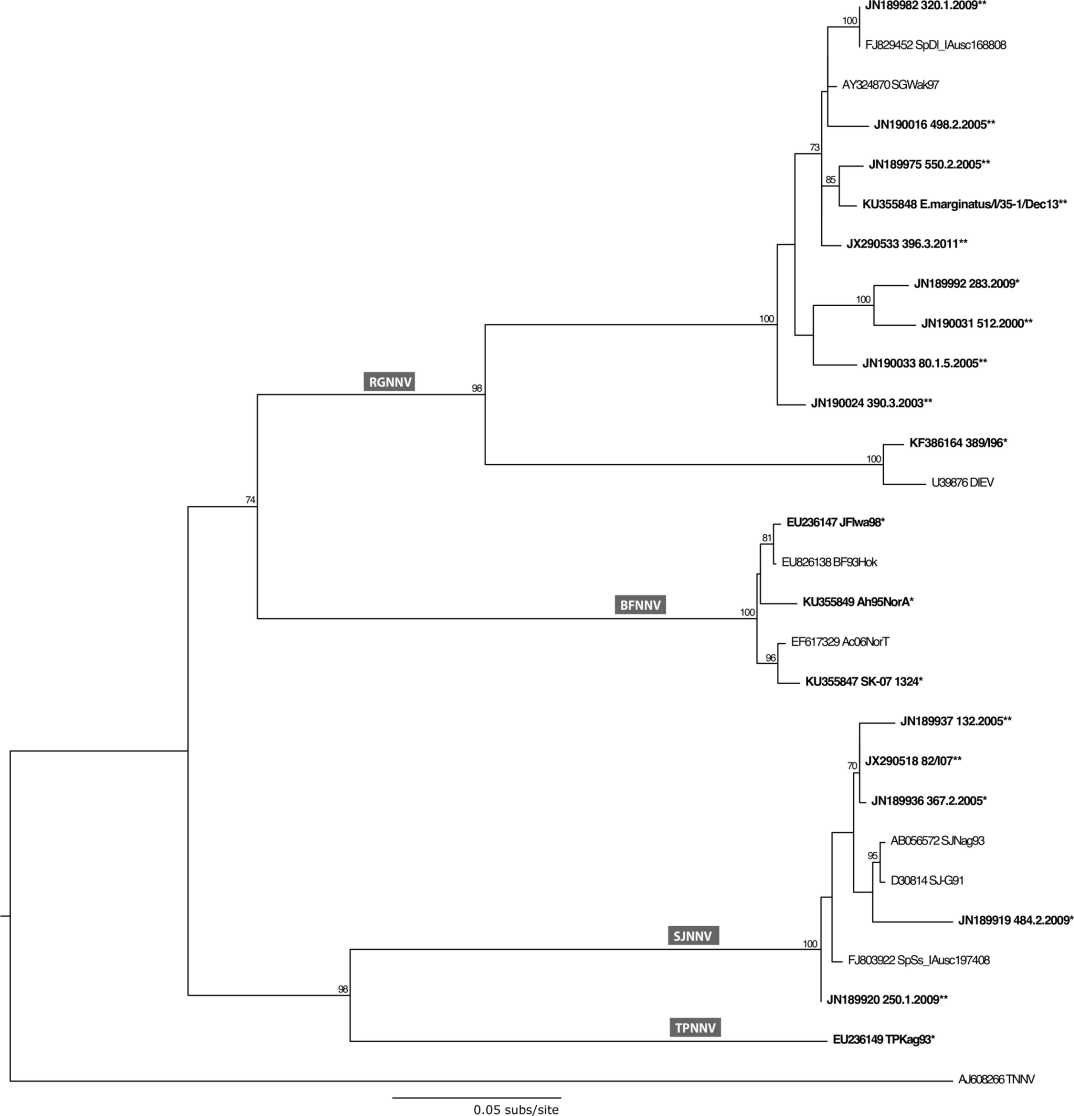
Phylogenetic tree of the betanodavirus isolates used in the present study. Partial RNA2 sequences related to the betanodaviral strains under investigation were aligned and compared with reference sequences available in GenBank. The phylogenetic tree was inferred using the maximum likelihood method (ML) available in the RaxML program, incorporating the GTR model of nucleotide substitution with the CAT model of rate heterogeneity among sites [48,49]. To assess the robustness of individual nodes, 100 bootstrap replicates were performed. Betanodavirus isolates used in the present study are highlighted in bold. *Viral isolates used for rabbit hyperimmune sera production and serological classification of fish nodaviruses. **Unknown field isolates used for blind evaluation of the SN test.

Eight betanodaviruses were used to produce rabbit immune antisera: 283.2009 (RGNNV), 484.2.2009 (SJNNV), 367.2.2005 (RGNNV/SJNNV), 389/I96 (SJNNV/RGNNV), TPKag93 (TPNNV), JFIwa98 (BFNNV), SK-07 1324 (BFNNV) and Ah95NorA (BFNNV) (S2 Table) [27,40–43]. All but strains TPKag93 and JFIwa98 originate from Europe. Strain 484.2.2009 was provided by Dr. Francesc Padrós (Universitat Autònoma de Barcelona) and isolates SK-07 1324 and Ah95NorA were supplied by Dr. Hilde Sindre (Norwegian Veterinary Institute). Additional 11 field viruses isolated from diseased fish (*n* = 8 RGNNV and *n* = 3 RGNNV/SJNNV) (S2 Table) [27,40,44] were serologically characterized blindly to evaluate the capability of the SN tests to serotype correctly. Viruses were selected to cover a wide variability in terms of host species, year of isolation, geographic origin and genetic features. The RNA2 sequences of the strains used in this work were retrieved from GenBank apart from SK-07 1324, Ah95NorA and E.marginatus/I/35-1/Dec13, which were obtained in the present study according to Panzarin et al. [45]. Pairwise nucleotide distances estimated with MEGA5 [46] and the phylogenetic tree describing the genetic relationships existing between all 19 viral strains are given in supplementary material (S3 Table) and in Fig 4, respectively.

The viruses were propagated in E-11 cells and after completion of cytopathic effect (CPE) media were collected, clarified at 4400 × *g* (Beckman centrifuge JA-21, rotor JA-10) for 60 min at 4°C and titrated. Viral titres, expressed as TCID_50_/ml, were calculated according to the Spearman-Kärber formula [47].

The viral antigens for rabbit immunization were purified by precipitation by adding 8% w/v poly-ethylene glycol (BioUltra 8 000, Sigma-Aldrich) and 2.2% w/v NaCl and incubated at 4°C overnight with gentle shacking. Samples were then centrifuged at 4400 × *g* for 120 minutes at 4°C, the pellets were dissolved 1:100 in TEN buffer (Tris-HCl 40 mM; EDTA 1 mM; NaCl 150 mM, pH 7.6), and sonicated 5×30 seconds on ice. Viral suspension was layered on a caesium chloride continuous gradient (Sigma-Aldrich) (27–40% w/v) and centrifuged for 18 hours, 110000 × *g*, at 4°C (Beckman centrifuge L-100K, rotor SW41Ti). Virus bands were collected with a syringe and then precipitated by centrifugation at 4°C for 4 hours at 110000 × *g*. Pellets were dissolved overnight at 4°C in 1 to 3 ml of TEN buffer. Purified viruses were titrated in E-11 cells and final viral titres were determined as described above.

### Rabbit antisera production

Antisera were obtained by immunizing six month-old New Zealand rabbits with subcutaneously injection of 0.3 ml of purified virus inactivated with 1% of formalin and emulsified with 0.7 ml of adjuvant Montanide ISA763VG (Seppic). This treatment was followed by 5 to 6 administrations of 0.5 ml live purified virus (without adjuvant) in the lateral ear vein. Inoculations were made at time intervals of 3 weeks. Local anaesthetic (lidocaine) was applied 20 minutes before each injection. Seroconversion was checked after the fifth intravenous injection, and when satisfactory titres were achieved (i.e. serum neutralization titres >1:1280/2560), the rabbits were bled to death after deep anaesthesia (pre-anaesthesia with 2 mg/kg acepromazine; anaesthesia with 7 mg/kg xylazine and 50 mg/kg ketamine). The serum was harvested and clarified by centrifugation. Rabbit hyperimmune sera were heat inactivated at 56°C for 30 minutes, aliquoted and stored at -20°C until use.

### Ethic statement

The immunization procedure was approved by the Ethics Committee of the Istituto Zooprofilattico Sperimentale delle Venezie (IZSVe) (decision n° 3/2013 of the 13^th^ May 2013) and authorized by the Italian Ministry of Health (IZSVe protocol n° 0006230/2013 of the 19^th^ June 2013). The animal care and use protocol herein adopted adheres to the Directive 2010/63/EU of the European Parliament and of the Council, implemented at national level through the D. Lgs 4^th^ March 2014, n.26.

### Neutralization assays

To describe the serological relationship among betanodaviruses with diverse genomes, cross-SN tests were carried out in four independent replicates by testing each serum against each of the eight viruses used for immunization. Two-fold serial dilutions of serum (from 1:20 to 1:40960) were prepared in 96 well plates (Corning) with L-15 medium (Leibovitz) (Sigma-Aldrich) without FBS. Four parallel wells were used for each serum dilution. Diluted sera were incubated with 100 TCID_50_/25μl of viral suspension. After an overnight incubation at 4°C, the virus-serum mixture was inoculated onto confluent E-11 monolayers and incubated for 10 days at 25°C (for the SJNNV, RGNNV, RGNNV/SJNNV and SJNNV/RGNNV genotypes) or at 20°C (for the TPNNV and BFNNV genotypes). Every 3 days cells were scrutinized for CPE appearance. The neutralizing titres were determined as the reciprocal of the highest dilution of serum capable to neutralize the virus in at least two out of four wells. Neutralizing titres were transformed into Log2 values. The geometric mean titre (GMT) was calculated among the four replicates and the value obtained was converted again into neutralizing titre. The Archetti and Horsfall formula was applied to estimate the 1/r index which describes the serological relationships among different betanodavirus isolates [31]. This parameter derives from the function $\text{r}=\sqrt{{\text{r}}_{1}\times {\text{r}}_{2}}$, where r_1_ is calculated by dividing the heterologous titre obtained with virus 2 by the homologous titre obtained with virus 1, and r_2_ derives from the ratio between the heterologous titre obtained with virus 1 and the homologous titre obtained with virus 2. The 1/r parameter defines the extent to which two viruses are serologically correlated when both antigens and both antisera are tested in a cross-serological reaction. A 1/r value of 10 or higher indicates that two viruses belong to distinct serological groups, while a value equal to 1 implies no antigenic difference [32].

For blind evaluation of the SN assay, further 11 field strains were subjected to serological characterization by testing in single the whole panel of rabbit hyperimmune antisera following the procedure described above.

### Statistical analysis

The Spearman coefficient (*r*_s_), estimated on the Log2 neutralization titres, was used to evaluate the presence of serological correlation between two different viruses [50]. Spearman coefficient is a non-parametric rank statistic for measuring the strength of a monotonic relationship between paired data, without making any assumption about the frequency distribution of the variables. The correlation coefficient ranges between -1 and 1. Absolute values of *r*_s_ are interpreted as follows: 0.00–0.19 “very weak” correlation; 0.20–0.39 “weak” correlation; 0.40–0.59 “moderate” correlation; 0.60–0.79 “strong” correlation; 0.80–1.0 “very strong” correlation. Correlation coefficients with *p*-value < 0.05 were considered significant.

Furthermore, in order to identify groups of viruses showing analogous immunoreactivity profiles, the principal component analysis (PCA), based on the Log2 values of neutralizing titres, was also applied [51]. This analysis is a variable reduction technique that provides a new set of uncorrelated and ordered variables (i.e. principal components, PCs) which are a linear combination of optimally-weighted observed variables (i.e. viruses). The estimation of PCs is obtained by the eigenvalue decomposition of the variance/covariance matrix. The number of PCs were selected according to the Kaiser criterion (eigenvalue > 1) [52], the scree plot of the eigenvalues versus the number of the components, the proportion of variance for each component, the cumulative proportion of variance explained and the interpretability of the principal components.

The statistical analyses were carried out with the software SAS version 9.3 (SAS Institute, Cary, N.C.).

### Plasmids preparation

**Fig 5:**
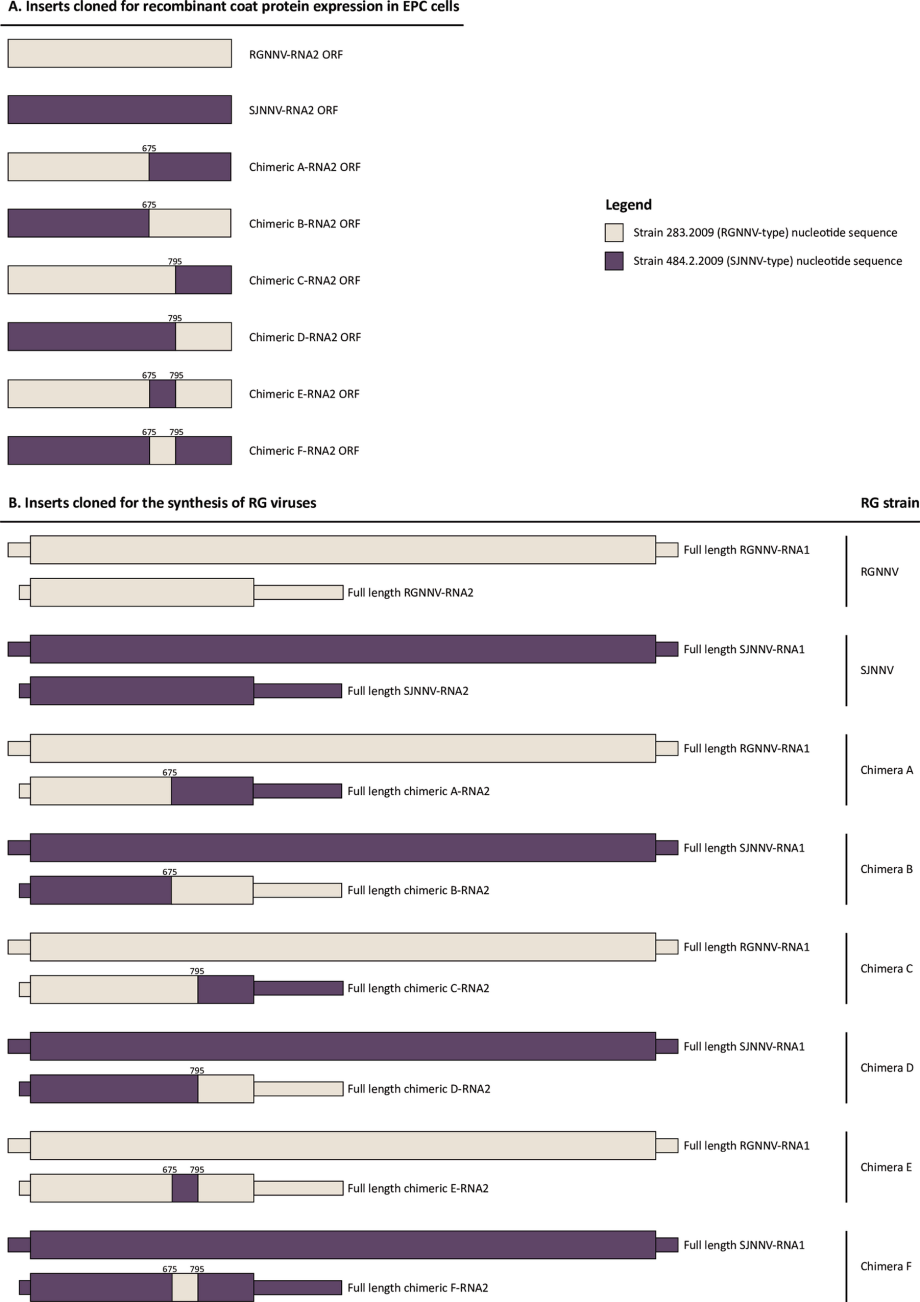
**Genetic structure of the RNA1 and the RNA2 inserts cloned for the expression of recombinant coat proteins in EPC cells (A) and for the generation of reverse genetics viruses (B).** Thick bars represent RNA1 and RNA2 open reading frames (ORF), and thin bars indicate the 5’-UTR and 3’-UTR regions. Light grey designates nucleotide sequence of the RGNNV genotype (strain 283.2009), while dark grey indicates genetic sequence of the SJNNV genotype (strain 484.2.2009). Numbers specify the nucleotide position of the chimeric junction between the RGNNV and the SJNNV sequences. Numbers refer to the full length RNA2 nucleotide sequence related to strain 484.2.2009 (GenBank accession number JN189919).

Total RNA was purified from 100 μl of clarified supernatants infected with strains 283.2009 (RGNNV) and 484.2.2009 (SJNNV) using the NucleoSpin RNA II (Macherey-Nagel GmbH & Co.). cDNA was synthetized with the SuperScript® III Reverse Transcriptase (Life Technologies) following the manufacturer’s instructions and by adding 10% DMSO to the reaction mix. Viral cDNA was amplified using specific primer sets (S4 Table) and the PfuUltra II Fusion HS DNA Polymerase (Agilent Technologies) according to the producer’s recommendations. The pcDNA^TM^3.1^(+)^ Mammalian Expression Vector (Life Technologies) was used to express wild-type and chimeric betanodavirus coat proteins in EPC cells under the control of cytomegalovirus (CMV) promoter. Chimeric open reading frame (ORF) sequences were synthetized by exchanging parts of the RNA2 segment between the RGNNV (283.2009) and the SJNNV (484.2.2009) strains (Fig 5A).

For the generation of reverse genetics viruses, the full length wild-type RNA1 and RNA2 sequences of isolates 283.2009 and 484.2.2009, as well as chimeric RNA2 genes engineered as above, were ligated into pI18 plasmid (kindly provided by Dr. Nigel Temperton) (Fig 5B). Cloning was carried out using the In-Fusion® HD Cloning Kit (Takara Bio Inc., Ōtsu, Japan) and the XL10-Gold® Ultracompetent Cells (Agilent Technologies) following the manufacturers’ instructions. The sequences of the DNA inserts were verified by sequencing.

### Recombinant capsid proteins expression and immunostaining

EPC cells were transfected with 2 μg of pcDNA™3.1^(+)^ plasmids containing wild-type and chimeric RNA2 ORF sequences (Fig 5A) using the Ingenio® Electroporation Solution (Mirus) and the Amaxa Nucleofector^TM^ Device (Lonza). After 24 hours incubation at 20°C, transfected cells were treated with 80% aceton. Fixated monolayers were then blocked with 10% foetal bovine serum (FBS) and subsequently immunostained for 1 hour at room temperature with rabbit polyclonal antibodies raised against betanodavirus strains 283.2009 (anti-RGNNV, diluted 1:20000) and 484.2.2009 (anti-SJNNV, diluted 1:3000). To avoid unspecific reactions, sera had previously been conditioned against EPC cells. Subsequently, washed cells were incubated for 45 min at room temperature with Alexa Fluor® 488 Goat Anti-Rabbit IgG (Life Technologies) diluted 1:1000. Finally, cells were added with a drop of Fluoroshield™ (Sigma-Aldrich), mounted with a cover glass and scrutinized under a fluorescence light microscope (Olympus IX81).

### Synthesis and characterization of reverse genetics viruses

Wild-type and chimeric RG betanodaviruses were generated by applying the principle of the infectious RNA transcription system developed by Iwamoto et al. [53]. Approximately 5 ng of pI18 plasmids containing the inserts of interest (Fig 5B) were digested at 37°C for 2 hours with *Eco*RI (New England Biolabs). Linear plasmids were subjected to electrophoresis in 1% agarose gel and were subsequently purified with the QIAquick Gel Extraction Kit (Qiagen) following manufacturer’s recommendations. About 1 μg of linear plasmid template was transcribed into RNA using the mMessage mMachine® T7 Transcription Kit (Life Technologies), which incorporates a cap analogue at the 5’ end of each genetic segment. The reaction mix was added with 40U of RNAsin® Plus RNase Inhibitor (Promega) and incubated at 37°C for 3 hours. The reaction was completed by 15 min incubation at 37°C with 1 μl TURBO DNase.

Synthetic RNA was purified using the MEGAclear^TM^ Transcription Clean-Up kit (Life Technologies) following the manufacturer’s recommendations. Twenty-four hours before transfection, E-11 cells (approximately 2.5 × 10^4^/ml) were seeded in 24-well plates with complete medium. A total of 500 ng of RNA1 and 250 ng of RNA2 transcripts were transfected into E-11 cells with the Lipofectamine® MessengerMAX™ Transfection Reagent (ThermoFisher Scientific) according to the producer’s instructions. Cells were incubated at 25°C and checked daily for CPE appearance. Upon disruption of cell monolayers, culture fluids were collected, frozen and thawed, and then passaged into fresh E-11 cells. After complete CPE had occurred and viral progeny had multiplied, cell culture supernatants were subjected to transmission electron microscopy (TEM). Samples were prepared according to standard procedures, negative stained with 2% sodium phosphotungstate solution and finally observed with transmission electron microscope (Philips 208S) [54]. Reverse genetics viruses were subjected to sequencing and serologically characterized through SN in four independent replicates as described above, using sera anti-RGNNV and anti-SJNNV. Their neutralization titres were compared with those of wild-type RGNNV (283.2009) and SJNNV (484.2.2009) strains.

## Supporting Information

S1 Fig PCA scree plot and explained variance. The scree plot on the left shows the eigenvalues versus the number of the components. The point where the slope of the curve is levels off (the “elbow”) indicates the number of components that have to be considered. The first three eigenvalues are identified and they are largely over 1. On the right, the plot shows that nearly the 92% of the total variance can be explained with the first three principal components.
(TIF)

S1 Table Serological characterization of betanodaviruses with different genomes. Cross-serum neutralization assays were performed by testing rabbit antisera against the selected antigens in four independent replicates. Neutralization titres are reported for each replicate. Antisera specificity was assessed also against viruses other than betanodavirus (i.e. VHSV and IHNV) in two independent replicates.
(DOCX)

S2 Table List of betanodavirus strains used in the present study. *Viral isolates used for rabbit hyperimmune sera production and serological classification of fish nodaviruses. **Unknown field isolates used for blind evaluation of the SN test.
(DOCX)

S3 Table Pairwise nucleotide distances estimated among the RNA2 sequences of the betanodaviral strains used in the present study. (DOCX)

S4 Table Primers adopted for the In-Fusion reactions. The RGNNV and the SJNNV specific sequences are underlined with dashed line and double line, respectively. The T7 promoter sequence is indicated in lowercase. Restriction enzyme sites are showed in italics (*Bam*HI: GGATCC; *Eco*RI: GAATTC; *Kpn*I: GGTACC; *Bgl*II: AGATCT). pcDNA^TM^3.1^(+)^ and pI18 specific sequences are indicated with continuous line and dot dash line, respectively.
(DOCX)
