# Supplementary figures and images for: Molecular Basis for Antigenic Diversity of Genus Betanodavirus
Source: PLoS One. 2016 Jul 20;11(7):e0158814. doi: 10.1371/journal.pone.0158814 (PMC4954670; doi:10.1371/journal.pone.0158814)

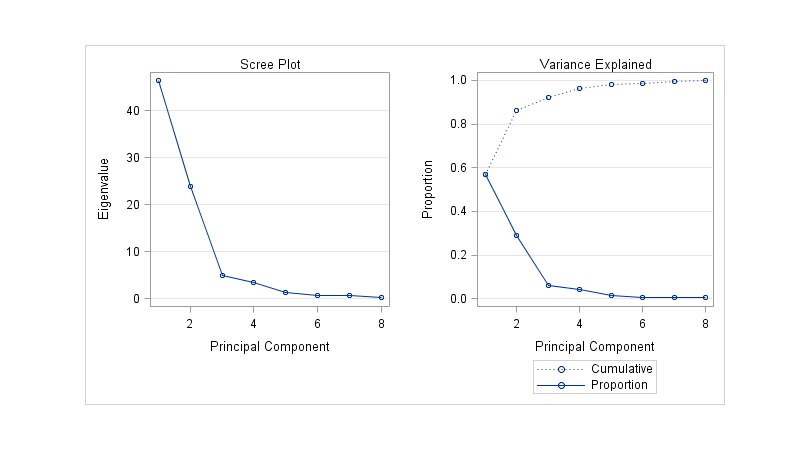

Supplement: S1 Fig — The scree plot on the left shows the eigenvalues versus the number of the components. The point where the slope of the curve is levels off (the “elbow”) indicates the number of components that have to be considered. The first three eigenvalues are identified and they are largely over 1. On the right, the plot shows that nearly the 92% of the total variance can be explained with the first three principal components. (TIF) [file pone.0158814.s001.tif]
