# Supplementary material for: Molecular Basis for Antigenic Diversity of Genus Betanodavirus
Source: PLoS One. 2016 Jul 20;11(7):e0158814. doi: 10.1371/journal.pone.0158814 (PMC4954670; doi:10.1371/journal.pone.0158814)
Supplement: S1 Table — Cross-serum neutralization assays were performed by testing rabbit antisera against the selected antigens in four independent replicates. Neutralization titres are reported for each replicate. Antisera specificity was assessed also against viruses other than betanodavirus (i.e. VHSV and IHNV) in two independent replicates. (DOCX) [file pone.0158814.s002.docx]

|  | Anti-283.2009 | | Anti-389/I96 | | Anti-484.2.2009 | | Anti-367.2.2005 | | Anti-JFIwa98 | | Anti-TPKag93 | | Anti-SK-07 1324 | | Anti-Ah95NorA | |
| --- | --- | --- | --- | --- | --- | --- | --- | --- | --- | --- | --- | --- | --- | --- | --- | --- |
| Virus | Rep. | SN titre | Rep. | SN titre | Rep. | SN titre | Rep. | SN titre | Rep. | SN titre | Rep. | SN titre | Rep. | SN titre | Rep. | SN titre |
| 283.2009 | 1 | 1:10240 | 1 | 1:640 | 1 | 1:40 | 1 | 1:320 | 1 | 1:10 | 1 | 1:80 | 1 | 1:10 | 1 | 1:40 |
|  | 2 | 1:20480 | 2 | 1:640 | 2 | 1:40 | 2 | 1:320 | 2 | 1:10 | 2 | 1:80 | 2 | 1:10 | 2 | 1:40 |
|  | 3 | 1:10240 | 3 | 1:1280 | 3 | 1:10 | 3 | 1:80 | 3 | 1:10 | 3 | 1:80 | 3 | 1:10 | 3 | 1:40 |
|  | 4 | 1:10240 | 4 | 1:1280 | 4 | 1:10 | 4 | 1:80 | 4 | 1:10 | 4 | 1:40 | 4 | 1:10 | 4 | 1:40 |
| 389/I96 | 1 | 1:20480 | 1 | 1:20480 | 1 | 1:40 | 1 | 1:320 | 1 | 1:10 | 1 | 1:80 | 1 | 1:10 | 1 | 1:80 |
|  | 2 | 1:10240 | 2 | 1:10240 | 2 | 1:10 | 2 | 1:80 | 2 | 1:10 | 2 | 1:40 | 2 | 1:10 | 2 | 1:20 |
|  | 3 | 1:20480 | 3 | 1:20480 | 3 | 1:10 | 3 | 1:80 | 3 | 1:10 | 3 | 1:80 | 3 | 1:10 | 3 | 1:80 |
|  | 4 | 1:10240 | 4 | 1:2560 | 4 | 1:10 | 4 | 1:160 | 4 | 1:80 | 4 | 1:80 | 4 | 1:80 | 4 | 1:80 |
| 484.2.2009 | 1 | 1:2560 | 1 | 1:320 | 1 | 1:5120 | 1 | 1:10240 | 1 | 1:10 | 1 | 1:40 | 1 | 1:10 | 1 | 1:10 |
|  | 2 | 1:2560 | 2 | 1:80 | 2 | 1:5120 | 2 | 1:10240 | 2 | 1:10 | 2 | 1:40 | 2 | 1:10 | 2 | 1:10 |
|  | 3 | 1:1280 | 3 | 1:160 | 3 | 1:20480 | 3 | 1:10240 | 3 | 1:10 | 3 | 1:40 | 3 | 1:10 | 3 | 1:10 |
|  | 4 | 1:1280 | 4 | 1:160 | 4 | 1:20480 | 4 | 1:20480 | 4 | 1:10 | 4 | 1:40 | 4 | 1:10 | 4 | 1:20 |
| 367.2.2005 | 1 | 1:640 | 1 | 1:160 | 1 | 1:2560 | 1 | 1:10240 | 1 | 1:10 | 1 | 1:20 | 1 | 1:10 | 1 | 1:10 |
|  | 2 | 1:320 | 2 | 1:80 | 2 | 1:2560 | 2 | 1:40960 | 2 | 1:10 | 2 | 1:20 | 2 | 1:10 | 2 | 1:10 |
|  | 3 | 1:1280 | 3 | 1:640 | 3 | 1:20480 | 3 | 1:40960 | 3 | 1:10 | 3 | 1:20 | 3 | 1:10 | 3 | 1:10 |
|  | 4 | 1:640 | 4 | 1:320 | 4 | 1:10240 | 4 | 1:40960 | 4 | 1:10 | 4 | 1:20 | 4 | 1:10 | 4 | 1:10 |
| JFIwa98 | 1 | 1:2560 | 1 | 1:160 | 1 | 1:80 | 1 | 1:320 | 1 | 1:640 | 1 | 1:2560 | 1 | 1:80 | 1 | 1:1280 |
|  | 2 | 1:1280 | 2 | 1:80 | 2 | 1:80 | 2 | 1:160 | 2 | 1:1280 | 2 | 1:2560 | 2 | 1:80 | 2 | 1:1280 |
|  | 3 | 1:2560 | 3 | 1:160 | 3 | 1:160 | 3 | 1:160 | 3 | 1:640 | 3 | 1:2560 | 3 | 1:320 | 3 | 1:1280 |
|  | 4 | 1:2560 | 4 | 1:160 | 4 | 1:160 | 4 | 1:160 | 4 | 1:1280 | 4 | 1:2560 | 4 | 1:160 | 4 | 1:1280 |
| TPKag93 | 1 | 1:20480 | 1 | 1:1280 | 1 | 1:1280 | 1 | 1:5120 | 1 | 1:2560 | 1 | 1:2560 | 1 | 1:1280 | 1 | 1:1280 |
|  | 2 | 1:640 | 2 | 1:1280 | 2 | 1:640 | 2 | 1:1280 | 2 | 1:2560 | 2 | 1:2560 | 2 | 1:2560 | 2 | 1:1280 |
|  | 3 | 1:320 | 3 | 1:160 | 3 | 1:320 | 3 | 1:1280 | 3 | 1:5120 | 3 | 1:5120 | 3 | 1:1280 | 3 | 1:2560 |
|  | 4 | 1:640 | 4 | 1:1280 | 4 | 1:640 | 4 | 1:1280 | 4 | 1:2560 | 4 | 1:2560 | 4 | 1:1280 | 4 | 1:1280 |
| SK-07 1324 | 1 | 1:20480 | 1 | 1:5120 | 1 | 1:1280 | 1 | 1:5120 | 1 | 1:10240 | 1 | 1:5120 | 1 | 1:10240 | 1 | 1:2560 |
|  | 2 | 1:5120 | 2 | 1:320 | 2 | 1:320 | 2 | 1:640 | 2 | 1:640 | 2 | 1:2560 | 2 | 1:320 | 2 | 1:640 |
|  | 3 | 1:10240 | 3 | 1:320 | 3 | 1:160 | 3 | 1:640 | 3 | 1:320 | 3 | 1:5120 | 3 | 1:640 | 3 | 1:2560 |
|  | 4 | 1:20480 | 4 | 1:640 | 4 | 1:640 | 4 | 1:5120 | 4 | 1:2560 | 4 | 1:5120 | 4 | 1:1280 | 4 | 1:1280 |
| Ah95NorA | 1 | 1:1280 | 1 | 1:320 | 1 | 1:640 | 1 | 1:640 | 1 | 1:80 | 1 | 1:1280 | 1 | 1:40 | 1 | 1:640 |
|  | 2 | 1:2560 | 2 | 1:1280 | 2 | 1:2560 | 2 | 1:5120 | 2 | 1:160 | 2 | 1:2560 | 2 | 1:80 | 2 | 1:1280 |
|  | 3 | 1:2560 | 3 | 1:1280 | 3 | 1:2560 | 3 | 1:2560 | 3 | 1:320 | 3 | 1:5120 | 3 | 1:320 | 3 | 1:5120 |
|  | 4 | 1:1280 | 4 | 1:640 | 4 | 1:1280 | 4 | 1:1280 | 4 | 1:80 | 4 | 1:2560 | 4 | 1:80 | 4 | 1:2560 |
| VHSV  Strain F-25* | 1 | 1:10 | 1 | 1:10 | 1 | 1:10 | 1 | 1:10 | 1 | 1:10 | 1 | 1:10 | 1 | 1:10 | 1 | 1:10 |
|  | 2 | 1:10 | 2 | 1:10 | 2 | 1:10 | 2 | 1:10 | 2 | 1:10 | 2 | 1:10 | 2 | 1:10 | 2 | 1:10 |
| IHNV  Strain 217/A** | 1 | 1:10 | 1 | 1:10 | 1 | 1:10 | 1 | 1:10 | 1 | 1:10 | 1 | 1:10 | 1 | 1:10 | 1 | 1:10 |
|  | 2 | 1:10 | 2 | 1:10 | 2 | 1:10 | 2 | 1:10 | 2 | 1:10 | 2 | 1:10 | 2 | 1:10 | 2 | 1:10 |

* DeKinkelin P, Bearzotti M. Immunization of rainbow trout against viral haemorrhagic septicaemia (VHS) with a thermoresistant variant of the virus. Dev Biol Stand 1981;49:431–9.

** Bovo G, Giorgetti G, Jorgensen P, Olesen NJ. Infectious haematopoietic necrosis: first detection in Italy. Bulletin of the European Association of Fish Pathologists 1987;7:124
