# Supplementary material for: Molecular Basis for Antigenic Diversity of Genus Betanodavirus
Source: PLoS One. 2016 Jul 20;11(7):e0158814. doi: 10.1371/journal.pone.0158814 (PMC4954670; doi:10.1371/journal.pone.0158814)
Supplement: S2 Table — *Viral isolates used for rabbit hyperimmune sera production and serological classification of fish nodaviruses. **Unknown field isolates used for blind evaluation of the SN test. (DOCX) [file pone.0158814.s003.docx]

| Strain | Genotype | Host | Origin | RNA2 Acc. No. | Reference |
| --- | --- | --- | --- | --- | --- |
| 283.2009* | RGNNV | *Dicentrarchus*  *labrax* | Italy | JN189992 | [27] |
| 484.2.2009* | SJNNV | *Solea*  *senegalensis* | Spain | JN189919 | [27] |
| 367.2.2005* | RGNNV/SJNNV | *Dicentrarchus*  *labrax* | Italy | JN189936 | [27] |
| 389/I96* | SJNNV/RGNNV | *Dicentrarchus*  *labrax* | Italy | KF386164 | [40] |
| JFIwa98* | BFNNV | *Paralichthys*  *olivaceus* | Japan | EU236147 | [41] |
| TPKag93* | TPNNV | *Takifugu*  *rubripes* | Japan | EU236149 | [41] |
| SK-07 1324* | BFNNV | *Gadus*  *morhua* | Norway | KU355847 | [42] |
| Ah95NorA* | BFNNV | *Hippoglossus*  *hippoglossus* | Norway | KU355849 | [43] |
| 512.2000** | RGNNV | *Dicentrarchus*  *labrax* | Italy | JN190031 | [27] |
| 390.3.2003** | RGNNV | *Solea solea* | Italy | JN190024 | [27] |
| 80.1.5.2005** | RGNNV | *Dicentrarchus labrax* | Italy | JN190033 | [27] |
| 498.2.2005** | RGNNV | *Mullus barbatus* | Italy | JN190016 | [27] |
| 550.2.2005** | RGNNV | *Epinephelus spp.* | Greece | JN189975 | [27] |
| 320.1.2009** | RGNNV | *Dicentrarchus labrax* | Spain | JN189982 | [27] |
| 396.3.2011** | RGNNV | *Epinephelus costae* | Italy | JX290533 | [44] |
| E.marginatus/I/35-1/Dec13** | RGNNV | *Epinephelus marginatus* | Italy | KU355848 | This study |
| 132.2005** | RGNNV/SJNNV | *Dicentrarchus labrax* | Italy | JN189937 | [27] |
| 82/I07** | RGNNV/SJNNV | *Sparus aurata* | Italy | JX290518 | [40] |
| 250.1.2009** | RGNNV/SJNNV | *Sparus aurata* | Cyprus | JN189920 | [27] |

*Viral isolates used for rabbit hyperimmune sera production and serological classification of fish nodaviruses

**Unknown field isolates used for blind evaluation of the SN test
