# Supplementary material for: Molecular Basis for Antigenic Diversity of Genus Betanodavirus
Source: PLoS One. 2016 Jul 20;11(7):e0158814. doi: 10.1371/journal.pone.0158814 (PMC4954670; doi:10.1371/journal.pone.0158814)
Supplement: S3 Table — (DOCX) [file pone.0158814.s004.docx]

|  | **283.2009** | **484.2.2009** | **367.2.2005** | **389/I96** | **JFIwa98** | **TPKag93** | **SK-07_1324** | **Ah95NorA** | **512.2000** | **390.3.2003** | **80.1.5.2005** | **498.2.2005** | **550.2.2005** | **320.1.2009** | **396.3.2011** | **E.marginatus/I/**  **35-1/Dec13** | **132.2005** | **82/I07** | **250.1.2009** |
| --- | --- | --- | --- | --- | --- | --- | --- | --- | --- | --- | --- | --- | --- | --- | --- | --- | --- | --- | --- |
| **283.2009** |  |  | | |  |  |  |  |  | |  |  |  | |  | | | | |
| **484.2.2009** | 0.22 |  |  |  |  |  |  |  |  |  |  |  |  |  |  |  |  |  |  |
| **367.2.2005** | 0.22 | 0.03 |  |  |  |  |  |  |  |  |  |  |  |  |  |  |  |  |  |
| **389/I96** | 0.16 | 0.22 | 0.22 |  |  |  |  |  |  |  |  |  |  |  |  |  |  |  |  |
| **JFIwa98** | 0.20 | 0.20 | 0.21 | 0.20 |  |  |  |  |  |  |  |  |  |  |  |  |  |  |  |
| **TPKag93** | 0.28 | 0.26 | 0.26 | 0.29 | 0.27 |  |  |  |  |  |  |  |  | |  |  |  |  |  |
| **SK-07_1324** | 0.19 | 0.21 | 0.22 | 0.20 | 0.02 | 0.21 |  |  |  | | | |  |  |  |  |  | |  |
| **Ah95NorA** | 0.18 | 0.21 | 0.22 | 0.19 | 0.02 | 0.21 | 0.02 |  |  |  |  | |  |  |  |  |  |  |  |
| **512.2000** | 0.03 | 0.23 | 0.22 | 0.16 | 0.21 | 0.22 | 0.19 | 0.21 |  |  |  |  |  |  |  |  |  |  |  |
| **390.3.2003** | 0.06 | 0.23 | 0.22 | 0.15 | 0.19 | 0.21 | 0.18 | 0.19 | 0.05 |  |  | |  |  |  |  |  | |  |
| **80.1.5.2005** | 0.05 | 0.23 | 0.22 | 0.15 | 0.19 | 0.22 | 0.19 | 0.19 | 0.05 | 0.03 |  |  |  |  |  |  |  |  |  |
| **498.2.2005** | 0.07 | 0.23 | 0.22 | 0.16 | 0.18 | 0.22 | 0.17 | 0.18 | 0.07 | 0.04 | 0.05 |  |  |  |  |  |  | |  |
| **550.2.2005** | 0.06 | 0.24 | 0.23 | 0.16 | 0.18 | 0.22 | 0.18 | 0.18 | 0.06 | 0.03 | 0.05 | 0.03 |  |  |  |  |  |  |  |
| **320.1.2009** | 0.07 | 0.22 | 0.22 | 0.16 | 0.17 | 0.22 | 0.18 | 0.17 | 0.07 | 0.04 | 0.05 | 0.02 | 0.03 |  |  |  |  |  |  |
| **396.3.2011** | 0.06 | 0.23 | 0.22 | 0.15 | 0.18 | 0.22 | 0.18 | 0.18 | 0.06 | 0.03 | 0.03 | 0.02 | 0.02 | 0.02 |  |  |  |  |  |
| **E.marginatus/I/35-1/Dec13** | 0.07 | 0.23 | 0.23 | 0.17 | 0.18 | 0.22 | 0.18 | 0.18 | 0.07 | 0.04 | 0.05 | 0.02 | 0.01 | 0.02 | 0.02 |  |  |  |  |
| **132.2005** | 0.22 | 0.05 | 0.01 | 0.25 | 0.24 | 0.20 | 0.24 | 0.24 | 0.22 | 0.22 | 0.22 | 0.22 | 0.22 | 0.22 | 0.22 | 0.23 |  |  |  |
| **82/I07** | 0.22 | 0.04 | 0.00 | 0.24 | 0.23 | 0.19 | 0.24 | 0.24 | 0.22 | 0.21 | 0.22 | 0.22 | 0.23 | 0.22 | 0.22 | 0.23 | 0.01 |  |  |
| **250.1.2009** | 0.22 | 0.04 | 0.01 | 0.25 | 0.23 | 0.19 | 0.23 | 0.23 | 0.22 | 0.21 | 0.22 | 0.21 | 0.22 | 0.21 | 0.22 | 0.23 | 0.02 | 0.01 |  |
