# Supplementary material for: Molecular Basis for Antigenic Diversity of Genus Betanodavirus
Source: PLoS One. 2016 Jul 20;11(7):e0158814. doi: 10.1371/journal.pone.0158814 (PMC4954670; doi:10.1371/journal.pone.0158814)
Supplement: S4 Table — The RGNNV and the SJNNV specific sequences are underlined with dashed line and double line, respectively. The T7 promoter sequence is indicated in lowercase. Restriction enzyme sites are showed in italics (BamHI: GGATCC; EcoRI: GAATTC; KpnI: GGTACC; BglII: AGATCT). pcDNATM3.1(+) and pI18 specific sequences are indicated with continuous line and dot dash line, respectively. (DOCX) [file pone.0158814.s005.docx]

| **Primer** | **Sequence 5’🡪3’** |
| --- | --- |
| **RNA1** | |
| pI18_RdRp_RG_5’UTR_F  pI18_RdRp_RG_3’UTR_R  pI18_RdRp_SJ_5’UTR_F  pI18_RdRp_SJ_3’UTR_R | TCCTTGACACGATC*GGATCC*taatacgactcactatagTAACATCACCTTCTTGCT  TGACGTGGGATCTA*GAATTC*GCCGAAGCGTAAGACAGCA  TCCTTGACACGATC*GGATCC*taatacgactcactatagTAACATCAGCTCTTGCTCTG  TGACGTGGGATCTA*GAATTC*GCCGAAGCGTAGGACAGCA |
| **RNA2** | |
| pcDNA3.1_RG_N-ter_F  pcDNA3.1_RG_C-ter_R  pcDNA3.1_SJ_N-ter_F  pcDNA3.1_484_C-ter_R  pI18_283_5’UTR_F  pI18_283_3’UTR_R  pI18_484_5’UTR_F  pI18_484_3’UTR_R  675_RG/SJjunct_R  675_RG/SJjunct_F  675_SJ/RGjunct_R  675_SJ/RGjunct_F  795_RG/SJjunct_R  795_RG/SJjunct_F  795_SJ/RGjunct_R  795_SJ/RGjunct_F | TTAAACTTAAGCTT*GGTACC*ATGGTACGCAAAGGTGAGAAGAAATTGG  ATCCGAGCTC*GGTACC*TTAGTTTTCCGAGTCAACCCTGGTGC  TTAAACTTAAGCTT*GGTACC*ATGGTACGCAAAGGTGATAAGAAATTGGCA  ATCCGAGCTC*GGTACC*TTAGTTTTCCGAGTCAACACGGGTGAAG  TACCTCTAGA*AGATCt*aatacgactcactatagTAATCCATCACCGCTTTGCAATCACAATG  GTGGGATCTA*GAATTC*GCCGAGTTGAGAAGCGATCAGC  TACCTCTAGA*AGATCt*aatacgactcactatagTAATCTAACACCGCTTTGCAAGTCAAAATGG  GTGGGATCTA*GAATTC*GCCGAGTATTGTAGCGATCAGCG  TGGAGCGGTGGTATCTTCAGGTGTTTCAAGAGACGGAACGC  CTTGAAACACCTGAAGATACCACCGCTCCAATCGCTAC  GGGAGCGGTGGTCTCCTCAGGTGTCTCGAGGGACG  CTCGAGACACCTGAGGAGACCACCGCTCCCATCATGA  AGTGACATAGACAGCTCCGTCAGGAGCAATGTCCAGT  ATTGCTCCTGACGGAGCTGTCTATGTCACTGACAAGCCGTT  CAGCTGGAAGATTGCGTTTGCAGGGGCGAGGTCG  CTCGCCCCTGCAAACGCAATCTTCCAGCTGGACCGT |
